# Osteoblastoma of the frontal bone invading the orbital roof: A case report

**DOI:** 10.1097/MD.0000000000012803

**Published:** 2018-10-19

**Authors:** Kun Wang, Feidan Yu, Keng Chen, Huanjiang Niu, Yirong Wang, Shuxu Yang, Xiujun Cai

**Affiliations:** a Department of Neurosurgery; b Department of Radiology, Hangzhou Xiasha Hospital; c Department of Neurosurgery; d Department of General Surgery, Sir Run Run Shaw Hospital, Medical College, Zhejiang University, Hangzhou, China.

**Keywords:** diagnosis, osteoblastoma, skull, treatment

## Abstract

**Rationale:**

Osteoblastoma is an uncommon primary bone tumor that involves any part of the skeleton. But its occurrence in the skull is extremely rare.

**Patients concerns:**

A 30-year-old female was admitted to our hospital, because of the mass in the right frontal region with the history of headache for 3 years without nausea or vomiting.

**Diagnosis:**

Initial differential diagnoses included hemangiopericytoma, atypical intraosseous meningioma, calvarial osteosarcoma, fibrous dysplasia, and histiocytosis, based on the results of enhanced CT and MRI.

**Interventions:**

A total surgical resection of the mass was performed.

**Outcomes:**

Postoperative histopathologic analysis demonstrated the typical features of osteoblastoma, the benign bone neoplasm. Serial radiologic examination did not show recurrence in the 6 months follow-up.

**Lessons:**

The radiologic appearance of the osteoblastoma is always confusing and makes the diagnosis difficult. We hope our case can give some clinical clues for the diagnosis and management of the disease.

## 1 Introduction

Osteoblastoma, a rare primary bone tumor, representing 0.8% to 1% of all bone tumors,^[1]^ was initially reported by Jaffe and Mayer in 1932,^[2]^ but the current term was first described in 1956 by Lichtenstein and Jaffe.^[3,4]^ Osteoblastoma usually affects the vertebral column and long tubular bones of the lower extremities.^[5]^ However, there are few reported cases of osteoblastoma affecting the skull.^[6,7]^ We describe a rare case of osteoblastoma located in the frontal region and orbital roof, with particular attention on the differential diagnosis and the treatment.

## 2 Case presentation

**Figure 1:**
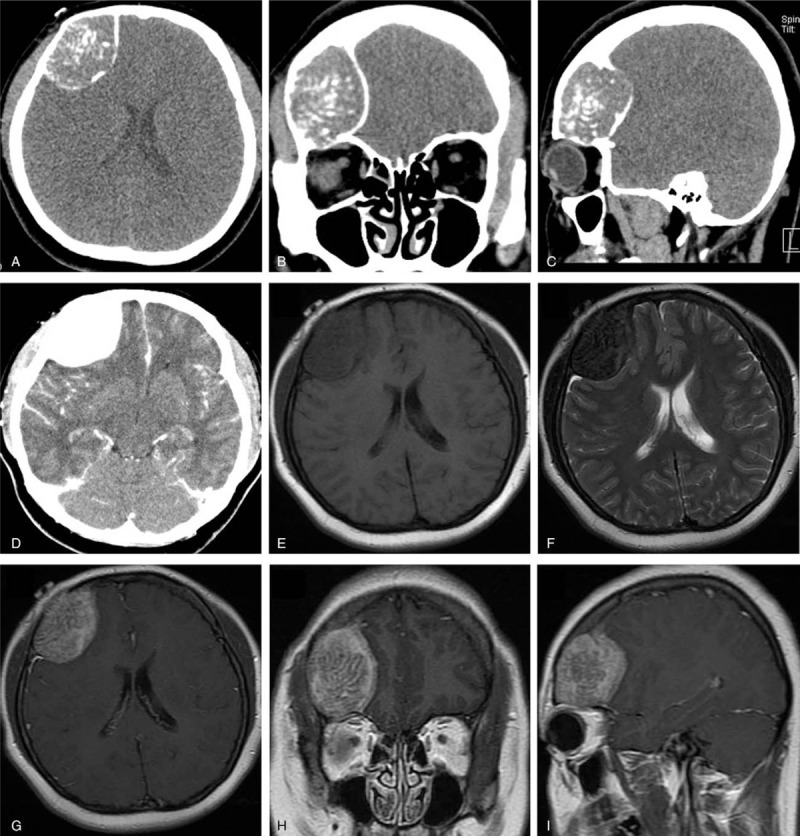
Preoperative computed tomography (CT) scan of the tumor in the frontal bone and orbital roof. (A–C) Preoperative CT of the head showed a 5 × 3.7 × 4 cm sized, well-demarcated osteolytic frontal lesion with mottled ossified density, which also infiltrated the orbital roof. (D) The result of enhanced CT, the lesion was shown to be with sufficient blood supply. (E–I) Enhanced magnetic resonance imaging magnetic resonance imaging revealed a solitary frontal mass, which was hypointense on T1- and T2-weighted images, and enhanced heterogeneously.

**Figure 2:**
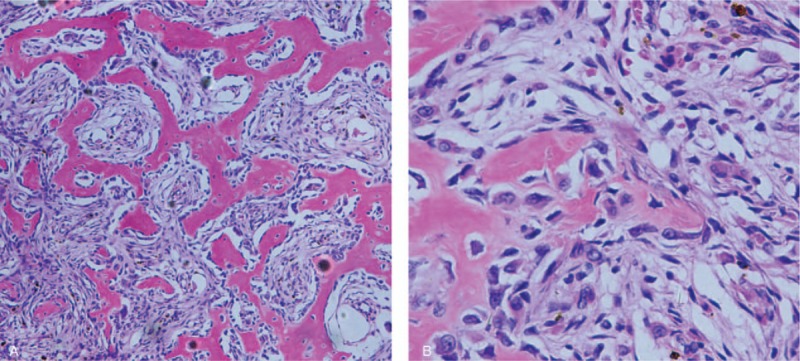
Pathologic appearance of the tumor. Hematoxylin and eosin (H&E) stained slide demonstrated a bone-forming tumor composed of diffusely trabeculae of woven bone, which was separated by richly vascular fibrous stroma, surrounded by osteoblasts. (A) 200×, (B) 400×.

A 30-year-old female, with a medical history of headache for 3 years, was referred to our department. Symptoms including dizziness, nausea, vomiting, limb movement disorder, or other neurologic deficits were not present in this case. But her headache has been growing progressively severe in the past 3 months. Physical examination demonstrated a palpable hard lesion of the right frontal region. Preoperative computed tomography (CT) of the head showed a 5 × 3.7 × 4 cm sized, well-demarcated osteolytic frontal lesion with mottled ossified density, which also infiltrated the orbital roof (Fig. 1A–C). Enhanced magnetic resonance imaging (MRI) revealed a solitary frontal mass, which was hypointense on T1- and T2-weighted images, and enhanced heterogeneously (Fig. 1E–I). It was also found in the images the brain distortion caused by mass effect of the lesion. Based on the results of enhanced CT and MRI (Fig. 1D, G–I), the lesion was shown to be with sufficient blood supply. Initial differential diagnoses included hemangiopericytoma, atypical intraosseous meningioma, calvarial osteosarcoma, fibrous dysplasia, and histiocytosis. Then the complete surgical removal was subsequently performed. Intraoperatively, a reddish-white mass, covered with a thin layer of bone and densely adhered to the underlying dura, was observed in the right frontal region. Postoperative histologic analysis demonstrated a bone-forming tumor composed of diffusely trabeculae of woven bone, which was separated by richly vascular fibrous stroma, surrounded by osteoblasts, which ultimately confirmed the diagnosis of osteoblastoma (Fig. 2). It was also observed in the stroma numerous thin-walled capillaries, occasional extravasation of blood and multinucleated giant cells. There was no radiologic evidence of recurrence in the 6 months follow-up.

## 3 Discussion

So far as we know, osteoblastoma is a rare primary bone tumor and occurs in any site of the skeleton and in all age groups, ranged from 4.5 months to 76 years.^[8]^ However, osteoblastoma rarely occurs in the skull, among which the most frequent site is the temporal bone.

The clinical symptoms are always nonspecific, including painful tumefaction, headache, visual disturbance, reduced auditory acuity, or facial paralysis.^[6]^ In our case, headache was the only symptom which was not sufficient for us to locate the lesion.

The radiologic features of osteoblastoma are always present as an intramedullary, well-demarcated, osteolytic lesion, averaging 4 cm in diameter with evidence of ossification and expansile behavior.^[6]^ The levels of ossification were closely related to pathologic stages of the disease and the patients’ age.^[9]^ Higher calcification and less lytic changes were commonly observed in the lesions of older patients. CT scans of the head usually demonstrate a well-demarcated sclerotic border with bony destruction, mottled calcification, and variable patterns with contrast enhancement.^[10,11]^ Computed tomography angiography or enhanced CT are used to determine the vascular supply of the tumor and adjacent soft tissues, which contributes to the surgical planning of the tumor. Osteoblastoma signal characteristics are variable on MRI, including isointense or hypointense signal on T1-weighted images and a hyperintense signal on T2-weighted images.^[12]^ Histologically, the most pathognomonic features are the presence of anastomosing trabeculae of osteoid and woven bone lined by a layer of activated osteoblasts.^[13]^

The differential diagnosis includes atypical intraosseous meningioma, calvarial osteosarcoma, fibrous dysplasia, histiocytosis and other benign osteogenic neoplasms, especially osteoid osteoma. Osteoid osteoma is typically <1.5 cm in diameter, while osteoblastoma is larger and rich in blood supply, which is a differential point on the basis of size.^[9]^ Clinically, the pain caused by osteoid osteoma can be relieved with nonsteroidal anti-inflammatory drugs (NSAIDs). In addition, osteoblastoma is often more aggressive and has a higher rate of recurrence than osteoid osteoma.

Complete resection is suggested due to the recurrence rate after incomplete resection appears to be up to 16% to 20% and the risk of malignant transformation.^[8]^ There is no evidence to support the benefit of radiotherapy or chemotherapy on osteoblastoma, except in recurrent or surgically unresectable patients.^[14]^

In summary, osteoblastoma usually involves any part of the skeleton as the primary bone tumor, while its occurrence in the skull is extremely rare. Our case showed a well-circumscribed osteolytic lesion with granular mineralization, and mottled ossified density at the right frontal bone involving the orbital roof, which was suspected as atypical intraosseous meningioma and fibrous dysplasia for the first time. Histologically, it demonstrated a vascular fibrous stroma with osteoid tissue or woven bone surrounded by osteoblasts. Though the radiologic appearance was sometimes misleading, the histopathologic result finally identified the diagnosis of osteoblastoma.

## Author contributions

**Conceptualization:** Kun Wang, Huanjiang Niu, Shuxu Yang.

**Data curation:** Feidan Yu, Keng Chen.

**Writing – original draft:** Kun Wang.

**Writing – review & editing:** Yirong Wang, Xiujun Cai, Shuxu Yang.
